# A dyadic approach to understanding the impact of breast cancer on relationships between partners during early survivorship

**DOI:** 10.1186/s12905-016-0337-z

**Published:** 2016-08-25

**Authors:** Sharon Keesing, Lorna Rosenwax, Beverley McNamara

**Affiliations:** 1 School of Occupational Therapy and Social Work, Curtin University, GPO Box U1987, Perth, WA 6845 Australia; 2 Deputy Pro Vice-Chancellor, Health Sciences, Curtin University, Perth, WA Australia; 3 Adjunct Professor, Curtin University, Perth, WA Australia

**Keywords:** Qualitative, Breast cancer, Survivor, Partner, Dyads, Relationships

## Abstract

**Background:**

The shared impact of breast cancer for women and their male partners is emerging as an important consideration during the experience of a breast cancer diagnosis, particularly during survivorship. This study aimed to explore the experiences of women and their partners during early survivorship and contributes a range of insights into the lives of those intimately affected by breast cancer.

**Methods:**

In-depth interviews were completed with Australian women survivors of breast cancer (*n* = 8) and their partners (*n* = 8), between six months and five years following cessation of treatment. Questions included a focus on the women and their partners’ daily experiences during early survivorship, including the management of ongoing symptoms, engagement in leisure and social interests, returning to work, communicating with each other, maintenance of the current relationship and other important roles and responsibilities. Thematic analysis was employed to determine key themes arising from the dyadic accounts of women and their partners’ experiences during early breast cancer survivorship.

**Results:**

Women and their partners experienced many changes to their previous roles, responsibilities and relationships during early breast cancer survivorship. Couples also reported a range of communication, intimacy and sexuality concerns which greatly impacted their interactions with each other, adding further demands on the relationship. Three significant themes were determined: (1) a disconnection within the relationship - this was expressed as the woman survivor of breast cancer needing to prioritise her own needs, sometimes at the expense of her partner and the relationship; (2) reformulating the relationship - this reflects the strategies used by couples to negotiate changes within the relationship; and (3) support is needed to negotiate the future of the relationship - couples emphasised the need for additional support and resources to assist them in maintaining their relationship during early survivorship.

**Conclusion:**

It can be concluded that the early survivorship period represents a crucial time for both women and their partners and there are currently limited options available to meet their shared needs and preferences for support. Findings indicate that a suitable model of care underpinned by a biopsychosocial framework, access to comprehensive assessment, timely support and the provision of targeted resources are urgently needed to assist women and their partners during this critical time.

## Background

Breast cancer is one of the most common cancers affecting women worldwide [1]. Advances in early detection and improved treatments have resulted in almost 90 % of women in Australia achieving a five-year survival [2]. The period following cessation of treatment, ‘survivorship’, is increasingly being recognised as an important time in the care of women diagnosed with this disease, due, in part, to the many physical, psychological and emotional sequelae of breast cancer [3]. In addition, the usual treatment regimes offered to manage breast cancer (e.g. surgery, chemotherapy, radiotherapy, adjuvant hormone therapy or combinations of these methods) can produce significant physical, psychological and emotional consequences for women in the longer term [4, 5].

Supportive care to address the consequences of breast cancer during survivorship has historically been focussed on the range of physical problems such as pain, lymphoedema, cognitive impairment, fatigue, premature menopause, sleep disturbances and other chronic health conditions [6, 7]. Also recognised are a range of psychological issues relating to a diagnosis of breast cancer including; changes in body image and self-identity, fear of recurrence, mood disturbances and significant disruption to activities, roles and relationships [8–11]. Internationally, targeted care during survivorship is increasingly recognised as critical to successful outcomes following a diagnosis of breast cancer. However, great diversity exists regarding service delivery models, the use of clinical guidelines, needs assessment tools, treatment summaries, survivorship care plans and care co-ordination [12–15]. Recent studies have also begun to explore other approaches to care including the use of self-management strategies, use of a chronic disease management approach, and the use of patient navigators [16–18]. Research findings indicate that a more focussed approach to comprehensive survivorship care is essential, with targeted interventions developed to address the unique and individual needs of women during this time [19–22]. Some progress has been made to evaluate the benefits of these interventions with promising results [23–26].

While the priority for survivorship care has been targeted towards women who are recovering from breast cancer, there is a recognition that the partners of women may also be considerably impacted by the experience of a breast cancer diagnosis [27–30]. Commonly reported concerns of partners during survivorship include a lack of information and education about survivorship, difficulty managing the expectations they have of themselves, difficulty coping with changes in the relationship with their partner, and problems re-adjusting to their previous role and responsibilities within the family [27, 31–33]. The shared experience of breast cancer may also create ongoing psychological issues for partners long after the cessation of treatment, including emotional withdrawal, guilt, anxiety, depression, difficulty communicating feelings of loss and grief, and fears of disease recurrence [5, 34, 35]. Partners may experience the same or higher levels of psychological distress as women and these may contribute to psychiatric issues in the longer term [36–38].

Supportive care which has focussed on the partners of women affected by breast cancer is predominantly confined to the period of diagnosis, treatment and end of life care [39–41]. However, several recent studies have explored the experiences and potential needs of partners during early survivorship [32, 38, 42, 43]. Women’s health and well-being can be significantly affected by their partner’s responses and unmet needs in the longer term [44, 45]. The potential for communication break-down, relationship worries and intimacy concerns between couples during survivorship is increased, hence it was found to be vital to consider the needs of both women and partners across the entire continuum of care, being diagnosis, treatment and survivorship [32, 46].

Cessation of treatment marks a milestone in the breast cancer journey yet many women report increased difficulties at this time as a result of less formal supports evident, fewer appointments with medical and health professionals, and the expectation that life will return to ‘normal’. Many women and partners find that life does not return to their previous level of function or routines and they are often unprepared for resultant changes [47, 48]. For women, the expectation is that their partner will move from caregiver and support person to their usual role, routines and responsibilities experienced before the breast cancer event. The literature reports inconsistencies regarding the impact of the breast cancer experience on relationships during this time. Some studies report an exacerbation of women and their partners’ existing problems relating to the expression of emotions, less open communication and changes in the usual resolution of problems, all of which may lead to increased stress and conflict [33, 49]. Partners may not respond in a helpful way due to their own distress, resulting in further communication and relationship difficulties [50, 51].

Conversely, several studies found that a diagnosis of breast cancer resulted in positive changes with couples becoming closer, perhaps from the development of sophisticated communication skills required to manage the challenges of the diagnosis and treatment [28, 52]. Our study supports recent literature which calls for further exploration of the interactions between women and their partners during diagnosis, treatment and survivorship to further understand this complex phenomenon [40, 53]. Specifically, we focus on the early survivorship period as there is a lack of strong evidence to understand the challenges experienced during this critical time. Some authors state that women and their partners should be considered as a ‘dyad’- with each person bringing their own experiences and coping strategies to the partnership, but with an interdependent approach to managing their relationship during survivorship [39, 53–57]. Our study suggests that the dyadic approach provides a comprehensive and in-depth view of survivorship and aims to extend the existing knowledge of this critical period for both women and their partners.

### Survivorship models of care and the Australian context

Since the publication of the Institute of Medicine’s (2006) report ‘From cancer patient to cancer survivor: lost in transition’, there has been an increased focus on research and evaluations dedicated to the improvement of models of care and guidelines, as well as services and tools directed to survivors of cancer worldwide [58, 59]. In Australia, there continues to be considerable variation regarding the models of care offered to women survivors of breast cancer. These include specialist (oncologist) led consultations, primary care (physician or general practitioner) led services, shared care (often using a clinic-based model) directed by nurses, and patient initiated models [60–62]. The range of services offered to women is also varied according to the preferred model of care, location, public versus private health service coverage and the availability of suitably experienced health professionals.

Consistency regarding the use of essential tools including survivorship care plans (SCP’s), treatment summaries and improved co-ordination of care is needed [62]. There are also limited available resources targeted towards the partners and families of women survivors of breast cancer during survivorship, with an increasing recognition that holistic models of care must consider the needs of partners and families when developing resources [63, 64]. In 2015, the Clinical Oncology Society of Australia (COSA) called for urgent attention to recognise the limitations of current survivorship practices and effect a range of improvements to survivorship care in Australia [65]. Further research must be directed towards improving the range of supports directed at partners and couples to address ongoing concerns.

### Aims

The aims of the study were to: identify changes in the way couples communicate with each other during early survivorship; determine the behaviours and actions used by women and their partners in maintaining their relationship during early survivorship; and identify the needs and supports required by women and their partners during early survivorship.

## Methods

The research used a dyadic interview methodology to explore and understand the experiences of couples during early survivorship of breast cancer. The use of dyadic interviews offers a range of benefits regarding the phenomena of concern [66–68]. Demonstration of both consensus and disparity between the interviewees, corroboration, improved levels of comfort and support between participants, observations regarding non-verbal behaviours, and a broader scope of the experience may all be evident using this technique [69]. The advantages of dyadic interviewing extend further to allow insight into how both individuals react and respond within the dyad, providing an alternative interpretation of the experience [70]. There are a growing number of studies utilising a dyadic approach to consider the experience of cancer for partners and spouses [39, 51, 71]. The benefits of a dyadic approach are relevant for exploring breast cancer survivorship, as during this period women and their partners usually need to negotiate and reconsider their previous relationship, routines and responsibilities. Dyadic interviewing has some potential disadvantages such as withholding of information due to the presence of an intimate partner, disagreement and interviewer bias [70]. The use of peer review, member checking and a reflexive journal were strategies used to minimise potential bias [72]. Well-developed interview techniques were also used to ensure each participant had adequate time to consider questions and acknowledge potential disagreements.

In-depth interviews of women (*n* = 8) and their partners (*n* = 8) were completed, with six couples interviewed together and the remaining two couples interviewed individually due to scheduling difficulties. All participants were asked to describe their experiences regarding diagnosis, treatment and survivorship of their (or their partner’s) breast cancer with particular emphasis on the period following cessation of treatment (early survivorship) [73]. In-depth interviews allowed the researcher to ‘have a conversation’, listen, understand and make sense of the participant’s experience of the phenomena they were describing [74, 75].

Interview questions were developed following review of the literature [76, 77]. The questions were further refined following a pilot completed with a non-participant couple. The first author commenced each interview with a series of demographic questions, including age, occupation, level of education and marital status, which assisted to build rapport with each participant. Demographic information is presented in Table 1. Open-ended questions about the women’s experiences during diagnosis and treatment were completed with prompting questions to target the thoughts and feelings of the particular period in the participants’ life [78]. Further questions were asked regarding their experiences following the conclusion of treatment and the transition to survivorship (Table 2).

**Table 1:** Demographics of women and men participants

Participant	Current age range (in years)	Education	Marital status	Parenting and number of children living at home	Partner interviewed separately	Date of diagnosis	Time since treatment completed	Treatment	Service type	Religious or cultural background
1	45–50	University Degree	Married	Yes/2	No	May 2011	3 years	Bilateral mastectomy, chemotherapy, hormone therapy, preventative hysterectomy, Breast reconstruction	Private	Nil identified
2	45–50	University Degree	Married							Nil identified
3	35–40	Year 12	Married	Yes/2	No	October 2012	1 year	Bilateral mastectomy, chemotherapy radiotherapy, hormone therapy, breast reconstruction	Private	Nil identified
4	30–35	Year 12	Married				10 months			Nil identified
5	40–45	University Degree	Married	No	Yes	April 2013	1 year 3 months	Unilateral lumpectomy, chemotherapy radiotherapy, hormone therapy	Private	Nil identified
6	45–50	University Degree	Married							Nil identified
7	45–50	Year 10	Married	Yes/1	No	May 2009	5 years	Unilateral lumpectomy, chemotherapy radiotherapy, hormone therapy	Public	Nil identified
8	45–50	Not known	Married							
9	50–55	Diploma	Married	Yes/2	Yes	August 2013	1 year	Unilateral lumpectomy, chemotherapy, radiotherapy, hormone therapy	Mix	Nil identified
10	50–55	University Degree	Married							Nil identified
11	50–55	University Degree	Married	Yes/0	No	October 2012	2 years	Unilateral lumpectomy, radiotherapy	Public	Jewish
12	50–55	Not known	Married							
13	45–50	Year 12	Married	Yes/1	No	July 2012	2 years	Bilateral lumpectomy, chemotherapy, Mastectomy, hormone therapy	Private	Nil identified
14	45–50	Year 12	Married				2 months			
15	50–55	University Degree	Married	Yes/2	No	February 2013	1 year	Unilateral lumpectomy, Chemotherapy, Unilateral mastectomy, hormone therapy	Mix	Nil identified
16	50–55	University Degree	Married				6 months

**Table 2:** Questions for women participants

1. What follow up care has been arranged for you e.g. doctor’s visits, tests, medication reviews?
2. What sort of ongoing problems or symptoms are you experiencing and how do you manage these?
3. What are the long term effects of the cancer/medications/treatment?
4. Were you given a survivorship care plan- what does it contain? Do you have a copy of it? How has it been used?
5. Has your life returned to the way it was previously? If not, how have your roles and responsibilities changed since finishing your cancer treatment?
6. Have your relationships with others (partner, family members) changed since the treatment finished? How?
7. What might be some potential positives to come out of the cancer experience?
8. Have you had any problems with resuming work? If not working, how do you spend your days currently?
9. Can you describe any resources or services that you are currently using and are these successful? Do you participate in a support group- what is this/is it effective for your needs? Are you satisfied with the resources and supports you are currently using- why/why not?
10. Do you feel that your partner is experiencing any issues following the completion of treatment? What are these?
11. What would your recommendations be for other cancer survivors?
12. Do you think that having cancer has changed you as a person and in what way?
13. How have your future plans and goals changed as a result of the cancer and or treatment?
Questions for partners
1. Now that treatment has finished for your partner, what’s your daily routine? How have your roles and responsibilities changed? Are you currently working? If not working, how do you currently spend your days?
2. Does your partner experience any ongoing problems or symptoms? Do these problems impact you and have you experienced any changes in your relationships with others (partner, family members) since the treatment finished?
3. Was your partner given a survivorship care plan- what does it contain? Do you have a copy of it?
4. Can you describe how the SCP has been used during this period? Was it utilised to identify any issues for you personally as well as your partner?
5. What might be some potential positives to come out of the cancer experience?
6. Has your life returned to the way it was previously, if not how has it changed?
7. Can you describe any supports that you are currently using (with or without your partner) and are these successful? Are you satisfied with the resources and supports you and your partner are currently using- why/why not?
8. Can you identify any needs that you personally feel have not been met?
9. Can you recommend any changes/improvements in services for the partners of cancer survivors?
10. Do you think that being the partner of a cancer survivor has changed you in any way? Have your future plans and goals changed as a result of the cancer and/or treatment?

Focus was directed towards the daily experiences of each participant including the management of ongoing symptoms, mood, engagement in leisure, hobbies and interests, social activities, returning to work, communication with others, relationships and current roles (parent, partner, worker and friend). The questions were rephrased for the woman’s partner. Each interview commenced with the woman initially and then moved to her partner; however, participants were invited to contribute at any stage of each other’s interview. Each interview (between 45 and 90 min per participant) was conducted by the first author face to face, recorded and transcribed using electronic media [79]. A numeric code and pseudonym was assigned to each participant to maintain confidentiality of data.

### Sampling and recruitment

Purposive methods were used to recruit eight women who identified as breast cancer survivors and their eight partners (all men); living in Perth, Western Australia [80]. Participant women were included if they met the stated inclusion criteria of age (35–70 years), had completed their treatment for breast cancer (excluding adjuvant hormone treatment) between six months and five years previously and spoke English. Purposive recruitment was identified as a strength to the study as this allowed women and their partners to offer their own unique perspectives on how the early survivorship experience affected their lives, both from the perspective of the individual as well as part of a dyad [81].

Potential women participants were excluded if they were receiving ongoing active treatment (e.g. surgery to remove a tumour, chemotherapy, radiotherapy) or palliative care. Participant women were recruited using a variety of strategies including written invitations on a network home page, community newspaper, local breast cancer network, community radio station and flyers posted on University noticeboards. Partners were invited to be interviewed if they identified as having an ongoing and significant relationship (married or defacto) with their wife/partner. They were asked to be involved in the study at the initial contact with participant women. All participants were provided with an information brochure outlining the purpose of the study, their time commitment to complete an interview on a voluntary basis, an assurance of confidentiality, benefits of the study, potential for discomfort and the opportunity to withdraw at any time.

Participants were also provided with information regarding telephone support services should they require assistance following completion of the interview, as it was acknowledged that some of the questions might have elicited negative memories regarding previous experiences. Interviews were conducted either in the participant’s home, place of work, or at the first author’s workplace. Written informed consent was obtained from all participants in the study. Ethical approval from the Human Research Ethics Committee of Curtin University was obtained prior to commencement of data collection (Approval number: HR 51/2014).

### Data analysis

Each transcript of the interviews was imported into NVivo © and this software was used to organise and categorise information from the participants. Thematic analysis was used to analyse the content of interviews using a six step process devised by Braun and Clark [82] and widely used in the qualitative literature. The first author read each of the transcripts line by line repeatedly to understand what was being stated by each of the respondents. The next step of the thematic analysis involved assigning a ‘description’ for each idea, event, reflection or phenomena discussed by the participant using an inductive approach [83]. These descriptions were then reviewed and further categorised into preliminary ‘themes’. Preliminary themes were refined across the three authors, provided with a defined title and finalised. Saturation of data was determined by the authors following this process as no new or emerging themes were discovered [74].

### Trustworthiness

Trustworthiness of the research was achieved using multiple methods. Peer review was utilised to discuss the development and progress of the research following interviews and during data analysis [84]. Member checking was used to confirm the authenticity of each transcript. Several participants made adjustments to the transcript following this opportunity. Memos and field notes were completed following each interview and contributed to the development of an audit trail [85].

## Results

A range of demographic similarities was evident among the group of participants. The mean age of women was 47 years (ranging from 38 to 52 years) and their partners 48 years (ranging from 34 to 53 years). All couples were married. Most women had secondary schooling and/or a university degree (*n* = 7). Similarly, six of the eight male participants had completed secondary schooling and/or a university degree. All women and their partners were currently working in paid employment. The mean time since completion of treatment was two years and two months, with a range of one year to five years.

Participant women and their partners spoke openly and in-depth about their experiences and challenges during survivorship, with three distinct themes established following analysis: (1) a disconnection within the relationship - this was expressed as the woman survivor of breast cancer needing to prioritise her own needs, sometimes at the expense of her partner and the relationship; (2) reformulating the relationship - this reflects the strategies used by couples to negotiate changes within the relationship; and (3) support is needed to negotiate the future of the relationship - couples emphasised the need for additional support and resources to assist them in maintaining their relationship during early survivorship.

The findings from this study support the extensive published literature regarding the physical and cognitive challenges experienced by women survivors of breast cancer. These included: changes to body image and identity, fatigue, sleep difficulties, pain, loss of range of movement in the affected limb, as well as a variety of cognitive symptoms including short term memory loss, concentration difficulties and poor motivation [86–91]. However, the use of a dyadic interviewing strategy presented a range of further issues impacting the relationships between women and their partners during early survivorship. The themes arising from these findings offer a unique ‘shared’ perspective of a couple’s experience during this time.

### A disconnection within the relationship

The first theme identified a range of personal and relationship changes experienced between couples during early survivorship. Most women reported that the experience of surviving breast cancer resulted in a need to always think of oneself and prioritise personal needs, before anyone else’s. They felt this changed the way they responded to others, especially their partner, which was often detrimental to the relationship. Describing this as a form of ‘selfishness’, coupled with her need for privacy, Fran (one year and three months post-treatment) describes her thoughts:

*I just want my space, I want a good night’s sleep …it’s just so important. I found that with breast cancer in one aspect it’s made me more selfish. I’m looking after myself rather than looking at whether he’s OK or not… I don’t really care whether you’re OK…I just want to take care that I’m OK.*

The period of early survivorship created a sense of disempowerment and women felt a need to regain control in the new environment of ‘survivorship’. Women stated that this resulted in a need to suppress their thoughts and feelings, as a strategy for coping with lost ‘control’. This might be explained as a form of ‘self-protection’ and resulted in an emotional disconnection with their partner and the relationship. Danielle (one year and ten months post-treatment) discusses further:

*I was so upset I would just yell at him and it was easy to throw my hands in the air…it’s not what I want to do but I’m not thinking straight, I’m not thinking like me*…*Our relationship has changed, it’s hard to know how to respond, it takes time to become yourself again, the expectation that things should be back to normal and it’s not. Learning to live afterwards is not as easy as what people presume it’s going to be. And trying to know what I want… every day was different. Poor David would never really have a clue… or what mood I’d wake up in or what I wanted or what I needed because it was different from yesterday*.

Partners also reported a range of difficulties when asked about their own experiences during early survivorship. Many stated that whilst they recognised and understood the many changes affecting their spouse, they felt that all they could do was observe and try not to react negatively to the situation. Some partners managed these difficulties by disregarding their own emotional needs, with an acceptance that the experience of breast cancer was continuing to impact their relationship long after cessation of treatment. It was also recognised that this sometimes meant a withdrawal from each other and resulted in the partner feeling rejected and isolated. A sense of detachment occurred creating further communication issues and limited opportunities for intimacy. Christopher (Carla’s partner) explains:

*We’ve had a lot of tough periods and I’m a caring person…I think the relationship issues that we’ve had in the last couple of years post cancer has sort of been around me being a bit detached… maybe that detachment is almost like that trauma kind of response… I’ve got to keep my distance here a little bit because there’s just so much going on and I don’t know how much more I can manage or deal with.*

When prompted to discuss the changes in their relationship during this period, many women recognised that their partner was withdrawing, but that were ill-prepared to provide support, due to their own adjustment difficulties. Partners also confirmed that they needed support during this time; however, they were unaware of where or how to obtain assistance. Marg (one year and six months post-treatment) describes the difficulties she experienced with her partner:

*My husband doesn’t talk about those sort of things and he deals with it by just doing practical things. He was very good that way but he didn’t share with me his concerns or what that could mean for the family. He was going through a difficult time at his work and I don’t think he felt supported well himself. People knew what was going on with me, I don’t think he really felt very well supported and it did affect him.*

Couples were very open with regard to the consequences of these communication difficulties and how their relationship was affected. It meant that they felt ‘stuck’ in their attempts to connect with each other, sometimes leading to conflict and stress. Some couples discussed many barriers regarding intimacy and resumption of sexual activity, a situation with which neither individual was satisfied. David (Danielle’s partner) and then Lara (two years and two months post-treatment) discuss further:

*We’ve been sort of non-intimate, I think it’s been once in two years. It messes with your brain because you start getting this thought that your partner doesn’t love you. Obviously you have different ideas about it and one of the doctors explained how it works with the female body…and to the point they sort of push you away.*

*They’re just a couple of lumps there…and I could have nipples put on but what would be the point? It’s not that there’s no point it’s just they still wouldn’t respond the way mine did … I want to feel the way I felt before but my body just isn’t the same and I felt a bit let down by my body… I am very hopeful that at some point I’ll feel more like me again. You know I haven’t totally written off our physical relationship.*

Changes to their communication with each other, continued stress, and a loss of intimacy during survivorship sometimes meant that couples’ future plans were very different to what they had anticipated prior to diagnosis. Christopher reflects on how the breast cancer experience impacted his relationship, resulting in changes to his thoughts about the future:

*The last couple of years have sort of been this rollercoaster of events… our way of coping and reactions and responses and that sort of thing… and it’s still going … I think it’s still going along in a way that’s sort of thrown us on a path that we wouldn’t have ordinarily been on perhaps. It’s led to us sort of drifting…drifting apart quite a bit…towards Carla’s kind of recovery phase and that led to a lot of questioning of where the relationship was at.*

### Reformulating the relationship

This theme reflects the opinions of women and partners regarding their attempts to accommodate changes in the relationship and the strategies they felt assisted them during early survivorship. Many women stated that their priority during early survivorship was to reclaim a sense of ‘self’ and that meant needing time and space for themselves before they could focus on the maintenance of their relationship. Women reported that a concentrated effort was required by their partners to understand and respect these needs, utilising open communication and empathy skills. Also recognised was that there were no clear answers about how long it would take to negotiate and adjust to the changes during this period. Marg explains her thoughts:

*You do feel like there’s some things I didn’t want to talk to or couldn’t talk to Matt about… It’s just, it’s happening to me and just have to sort it out and I knew that there was support all around me but there was just some things that I had to just do on my own and I thought at the end of the day yes it’s affecting everybody else but, I felt like it was happening just to me.*

When asked about their suggestions for managing the communication challenges in the relationship, many couples recognised that alternative solutions were needed. Communication styles that had worked previously were not always successful during early survivorship. Partners also commented on their role and capacity to support their spouse, given their own personal and emotional difficulties. Some partners stated that they were not always the first person that their spouse sought out for support, resulting in further frustration. David and Danielle describe how David’s usual actions and responses to his wife created problems for them:

I’m one of those people who love people to death you know what I mean? Like the big saying is love can fix anything, if it doesn’t work just increase the dose sort of thing… So that’s me in a nutshell and Danielle was sort of…. I need my space… and felt even though I’m away half the time from *FIFO (fly-in fly-out) she felt a bit smothered by it because I was always coming to her and so that’s my homework is for me to stay away and for her to come to me instead of the other way around.

*[Danielle interjects]: I think one of the biggest things, is that guys have to be very careful that they’re not doing things that benefit them. You know with the closeness thing, David would give me hugs, that’s what he actually needed at the time, it wasn’t what I needed… so it’s a very tough thing to learn.*

*FIFO- is the term coined to describe the work routine of individuals who need to be transported from their city of residence by aeroplane to place of work, often every 2–4 weeks throughout the year.

Women and their partners agreed that there were many challenges during this time; however, offered suggestions regarding potential ways to assist them in negotiating this new phase of their relationship. Couples agreed that they needed to acknowledge the communication issues, address their concerns together, and try to resolve these. A determined commitment to remain in the relationship was also articulated by women and partners with the view that progress would take time and patience from both parties. Lester (Lara’s partner) and Lara discuss their thoughts:

*It is a massive thing and that’ll be the show stopper for I’d say 60 – 70 % of marriages. It’s just that non-information and communication…can’t say more than stop the arguing side of things and talk and communicate what you’re actually trying to say. Don’t turn it into an argument, don’t storm out, just don’t.*

*I think it’s always been a big thing for us that we’d be there for each other no matter what… If you know that your partner’s going to be there no matter what, ‘cause there’s no one, there’s not a lot of people in this world for you.*

Many women reported that they were able to access a range of informal supports (friends, work colleagues, female family members) which greatly assisted them during early survivorship. These people were vital supports and offered women the opportunity to talk with someone other than their partner about their thoughts and feelings during this unpredictable period. This was in stark contrast to their partners’ experiences, and it was generally recognised that their partners often did not utilise their friends or family to discuss issues or concerns regarding the relationship. Couples agreed that both women and their partners needed someone to talk to away from each other and that this was very useful; offering another resource or just some time to listen to them during stressful periods. Glenda (five years post-treatment) and Gary (Glenda’s partner) share their experience:

*I used to say to Gary… he was in a club, building a hot rod at the time and I knew when the hammer got louder, he was taking his anger out on it and all the guys used to turn up and say ‘what do you want a hand with?’… I actually thought when they were all down the shed they’d be saying ‘how you going Gary?’ you know?*

*(Gary interjects) Blokes don’t talk to blokes like that you know… I mean you see these blokes’ sheds they’ve got if you’re depressed and things like that you know… Blokes… well blokes don’t talk about things like that.*

### Support is needed to negotiate the future of the relationship

Whereas the previous theme explored strategies that were used in an unplanned and ad hoc manner, the final theme identified the lack of a concrete plan which would enable the couples to move forward in a direct and co-ordinated manner. Women and their partners felt vulnerable and unprepared for this next stage in the breast cancer experience and were concerned about the future. The lack of a defined transition strategy, education and information about survivorship meant that many couples felt unprepared for this period, which also impacted their relationship with their partner. Danielle explains how this uncertainty affected her:

I *spoke to one of my doctors and he said to me when you were going through chemo and your treatment you wouldn’t have wanted me to say to you ‘you’re going to crash and burn afterwards’. He said ‘you wouldn’t want to hear that and you would have said no, I’m not’….. ‘So all we can do is wait for you to get to that and when you do we’re here’… But I think maybe, even if it was just information, even written, that you read in your own time when you’ve finished or maybe let you know that it is ok to feel like that. You know you may feel lost. No one really even said that. They’re just like oh… last treatment ‘See ya…’*

When asked about the supports and services accessed during early survivorship, women confirmed that these were more difficult to obtain compared with those sourced during treatment. None of the women interviewed were offered a survivorship care plan or written information following completion of treatment. Some women reiterated that often it was not until treatment had concluded, that many new fears and concerns emerged, especially regarding the resumption of previous roles and responsibilities. Also noted was the distinct lack of awareness regarding the potential for relationship difficulties. Ingrid (one year post-treatment), reported that she would have benefitted from ongoing support from the breast cancer nurse when her medical treatment was completed:

*That’s where I think the breast care nurse would have come in handier for me at the end of treatment… Not before and not during, at the end. Even if it was just a phone call or maybe you know… a visit or you can go there and see her.*

Couples were united in their suggestions about the potential value of formal support from health professionals to assist them in negotiating improvements in communication within their relationship. Some women stated that they had sought support to assist them with a range of personal and shared issues. Women and their partners recognised that ongoing communication difficulties could lead to long-term issues, including irreparable changes in the nature of their relationship and that professional assistance was needed to manage this. Marg discussed her strategy for addressing concerns:

*If you don’t recognise what’s happening and everything’s really, really hard then I think you need someone to help set you in the right direction. But if you’re aware enough, if things are pretty tough….go and see someone… which I was able to do. But I don’t know if everyone can do that and that was very difficult but it was very good in the long run.*

There was recognition that the partners of women were largely ignored with regard to requiring targeted support during the early survivorship period. Partners stated that they could have benefitted from a range of formal supports, but that they were not made aware of any potential resources during this period. Partners identified that access to support during this period was an initial step towards adjustment and gave hope for the future. Gary emphasises his desire for timely support to build a foundation towards a positive outcome following the cessation of treatment.

*I don’t think there’s enough for the guys, there’s more information for the women. But as far as information for guys…what to expect and how to cope with your wife … I mean fair enough because she’s the one going through it …but they don’t sort of scope on what happens around them… you really had no one to talk to. But it may be a few phone calls and a human face to face in private or whatever… then they might give you something and then that builds momentum. So something along those lines.*

## Discussion

The findings of this study support previously published literature regarding the experience of survivorship for women and raises many additional concerns about how the partners of women are also significantly impacted during this time. The physical and cognitive consequences of breast cancer and its treatments that continue during treatment and survivorship are well established and supported by many qualitative and quantitative studies [17, 59, 86, 92, 93]. Recent research focussing on the early transition from treatment to survivorship identifies further issues including loneliness and an inability to cope, as well as anxiety and emotional distress sometimes leading to depression [5, 11, 26, 92]. Our study’s findings raise additional concerns for women and their partners during survivorship, many of which have not been previously reported.

The women in this study expressed many psychological concerns relating to the early period following cessation of treatment. Women felt their physical, psychological and emotional needs were largely undervalued by their usual medical supports, with a sense that the psychological and emotional difficulties experienced during early survivorship were not considered a priority during this period. Women reported that the period immediately following cessation of treatment was the most problematic, with many emerging difficulties relating to the resumption of previous roles and responsibilities. A desire to ‘put themselves first’, a need for privacy, suppression of thoughts and feelings and being able to maintain control over their lives were frequently discussed as having a profound impact on daily function and maintenance of the relationship with their partner.

The resultant stress of coping with the diagnosis and treatment of breast cancer may be superseded by the problems experienced during survivorship [19, 47, 94–101]. There is some evidence to suggest that resources provided during this time including information, education, peer support/mentoring and self-management tools may assist women in preventing further issues including depression and other psychological sequelae [16, 102–104]. The literature reports that most women experience improvements in quality of life beyond the five year period (long–term survivorship) [105, 106]. However, some women do not; those with a previous history of depression and women who completed chemotherapy are thought to be at greater risk of long term problems [107, 108].

This study found the partners of women reported many unmet needs and were unaware of where they could obtain assistance to help them manage the many challenges experienced during survivorship, also citing that there was a lack of recognition for the important role they played in supporting their partner during this time. Common issues reported by participant partners of this study included; difficulty understanding and accommodating their partner’s needs during survivorship, communication issues, problems with intimacy and resumption of sexual activity as well as feeling isolated and detached from their relationship with their spouse. These findings add weight to the existing evidence that many partners feel largely unsupported during the breast cancer experience generally [21, 42, 92, 109]. While the supportive care efforts to meet partners’ needs appear to be improving, these are concentrated during the treatment period. Significant distress may continue beyond this the treatment period, resulting in further adjustment difficulties, anxiety and depression [21, 42, 51, 110, 111].

Results of this study reflect the complex interaction between women and their partners during survivorship and support the view that the breast cancer experience must be considered as ‘shared’. The literature describes this concept as a form of ‘dyadic coping’ and it explains the method for which women and partners learn the skills required to accommodate the stress experienced as a result of illness [39, 54]. This perspective is supported by several studies indicating that the psychological distress experienced by cancer survivors and their partners is interdependent with the recognition that cancer is a ‘family’ disease [27, 44, 54, 112, 113]. However, there are few Australian studies that highlight the unique needs of partners during breast cancer survivorship [114–116].

Changes to intimate relationships were also recognised by participants. Women reported that the physical and emotional changes experienced during survivorship resulted in them being unsure about if, and when, they would feel comfortable to resume a sexual relationship with their partner. Thematic findings of this research offer many examples of women needing to remain distanced from their partner, physically, emotionally and sexually. Treatment for breast cancer (chemotherapy, surgery and radiation) as well as the use of adjuvant (hormone) therapy are noted to potentially contribute to the physical and psychological consequences of breast cancer and may offer some explanation towards the complex relationship problems experienced at this time [32, 33, 117–119].

The findings of this study support the view that the experience of breast cancer for women with partners is profound. While the period of diagnosis and treatment is identified as creating significant stress for both partners regarding their relationship, emotional, financial and spiritual concerns [39], our findings indicate that the early survivorship period may continue to create many additional difficulties for couples. There is an expectation that women and their partners resume their previous activities and relationships with ease following cessation of treatment; however, the themes explored in this paper indicate that couples may experience a range of ongoing issues and partners themselves may have unique problems that are often overlooked.

There have been some efforts to date aimed at improving couples’ communication, coping skills and adjustment during survivorship. A systematic review conducted in 2013 [50] concluded that a range of psychological interventions completed with couples experiencing breast and gynaecological cancer were effective; however, the majority of the studies reviewed were focussed on the treatment stage [120–124]. Additional research has been completed examining a range of interventions applicable during survivorship including: adjustment to illness and the development of coping strategies [125]; addressing cancer related stress and improving marital communication [48]; and addressing body image concerns, intimacy and sexuality [126–128]. The findings of this study support the need for further development, application and evaluation of cost effective supports for couples affected by breast cancer, particularly during early survivorship.

Recommendations regarding the development, utilisation and efficacy of supportive care must be viewed within the context of that care. Models of cancer care vary considerably across the world, with a range of underpinning philosophies including a traditional medicalised approach, a biopsychosocial framework and the wellness model [65, 129–131]. To date, there is no consensus on which model/approach is most appropriate for the provision of support during breast cancer survivorship. It is widely recommended that these models of care must provide timely, cost effective services and supports according to the preferences of consumers [17]. Evidence from our study demonstrates that the experience of early survivorship for couples is complex, with many psychological, social and sexual issues, suggesting that a biopsychosocial framework is appropriate in addressing couples’ ongoing needs.

Researchers concur that there is an urgent need to further explore the efficacy of these approaches while observing the recommendations made by the Institute of Medicine (IOM), these being: the prevention of recurrent and new cancers; surveillance for cancer recurrence and medical and psychosocial late effects; strategies to manage the consequences of cancer; and co-ordination of specialists and primary providers [132]. The IOM also made key recommendations including the use of SCP’s to address many of the concerns raised by the current study’s participants [58]. There is a volume of literature suggesting that SCP’s may assist women to identify and address many of the ongoing issues and concerns relating to breast cancer survivorship [22, 25, 99, 133–135].

While participants of this study were not offered a SCP as a strategy for managing the consequences of breast cancer, the strongest recommendation of women and their partners was that they needed a formal plan to manage this new phase of their lives and to help them adjust to the many personal challenges being experienced during survivorship. Women and their partners interviewed in this study were left to negotiate this time on their own without the recommended supports and services needed to meet their varied and complex needs. Survivorship Care Plans may be an essential, yet underused resource that offers great potential to assist both women survivors and their partners to document, direct and facilitate the required supports and services in early survivorship. Our study suggests that any plan should also include the concerns of partners.

### Limitations

There are limitations to this research and some of these are common to the methodology of dyadic interviewing. Most women were interviewed with their partner present, which may have created a situation where a participant might not have offered information that could create difficulties or discomfort for the other person. Interestingly, the data gathered from the two couples interviewed separately from their partners were not dissimilar to that divulged by partners interviewed together. This may be because couples who agreed to be interviewed together felt comfortable in discussing their concerns with one another. It may be that the concerns of couples experiencing extreme distress may not be captured by this research project. All couples were heterosexual, limiting the unique perspectives that may have been provided regarding same sex couples. Inclusion criteria did not preclude same sex couples; however, no same sex couples volunteered for interviews.

Participants were asked about the support services utilised during survivorship; however, were not asked for their suggestions regarding potential recommendations, accessibility or the applicability of shared services and this is recognised as a limitation, warranting further investigation. Participant demographics indicate that the socio-economic status of couples was comparatively high and that the particular needs of individuals from low socio-economic groups were not represented in the findings. All participants were recruited from a large city and therefore may have been able to access services and supports if required. All participant women interviewed were married and therefore the findings may not be generalised to single/divorced women. However, it is reasonable to conclude that the findings may be transferrable to women residing in developed countries, where health and community services are comparable to existing Australian services.

## Conclusion

Results of this research support a shift from the traditional medicalised approach to a ‘biopsychosocial’ framework incorporating comprehensive multi-disciplinary care which targets women’s and their partner’s complex physical, psychological, communication and emotional needs, especially during early survivorship . Further development of this framework must complement the existing resources and be targeted towards the shared needs and preferences of women and their partners. Women survivors of breast cancer are recognised as a significant, yet distinct group of health care recipients requiring specialised and targeted services to manage their health care during survivorship. This paper provides additional evidence that the partners of women also experience a range of psychological, emotional and relationship consequences during survivorship. Women and their partners want increased awareness of, and support for, the important role partners provide during treatment and survivorship.

## Abbreviations

SCP: Survivorship care plan
